# An Essay on the Teeth

**Published:** 1849-07

**Authors:** 


*An Essay on the Teeth, by* A. Cook. Churchill, London, pp. 75.

We approve of the plan of writing books and pamphlets in a popular style for the perusal of non-professional persons, providing they contain timely scientific information and instructions upon a subject connected with the medical profession, as they not only tend to enlighten the public upon that in which they must feel great interest, and on which their health and comfort naturally depends.

It prevents their falling into the hands of the grossly ignorant and swindling quack, such as the Jones', the Thomas' and Rutters, alias, who not only *get the money,* but do irreparable injury to the profession. Many points treated in this pamphlet on the pathology of the teeth, are excellent, such as regards cleanliness and attention, in preventing the exciting cause of dental disease, in youth, the two crowded state of the teeth also is pointed out and scientifically treated. The work can be perused with much advantage to the general reader. R.

